# Transcriptome Analysis Reveals the Mechanism of Cold-Induced Sweetening in Chestnut during Cold Storage

**DOI:** 10.3390/foods13172822

**Published:** 2024-09-05

**Authors:** Chun Zhan, Ruqi Jia, Shuzhen Yang, Meihong Zhang, Litao Peng

**Affiliations:** College of Food Science and Technology, Huazhong Agricultural University, Wuhan 430070, China; chunzhan777@126.com (C.Z.); j15552677358@126.com (R.J.); yszhen@mail.hzau.edu.cn (S.Y.); zmh1576848384@163.com (M.Z.)

**Keywords:** chestnut, cold-induced sweetening, sucrose accumulation, starch degradation, transcriptomic sequencing, dormancy release

## Abstract

Chestnuts become sweetened with better tastes for fried products after cold storage, but the possible mechanism is not clear. The dynamics of sugar components and related physiological responses, as well as the possible molecular mechanism in chestnuts during cold storage, were investigated. Sucrose accumulation and starch degradation contributed to taste improvement. Sucrose content reached the peak after two months of cold storage, along with the accumulation of reducing sugars of maltose, fructose and glucose to a much lesser extent. Meanwhile, alpha-amylase and beta-amylase maintained high levels, and the activities of acid invertase and sucrose synthase increased. Transcriptome data demonstrated that differentially expressed genes (DEGs) were significantly enriched in the process of starch and sucrose metabolism pathway, revealing the conversion promotion of starch to sucrose. Furthermore, DEGs involved in multiple phytohormone biosynthesis and signal transduction, as well as the transcription regulators, indicated that sucrose accumulation might be interconnected with the dormancy release of chestnuts, with over 90% germinated after two months of cold storage. Altogether, the results indicated that cold storage improved the taste of chestnuts mainly due to sucrose accumulation induced by DEGs of starch and sucrose metabolism pathway in this period, and the sweetening process was interconnected with dormancy release.

## 1. Introduction

Chestnut (*Castanea mollissima* Blume) is one of the most important and popular types of nuts widely cultivated nut species, with a distribution spanning East Asia, Europe, and North America [1]. In China, chestnut has been cultivated for over 3000 years, with an output of 1.8 million tons in 2019, representing 75% of global production [2]. Recent investigations revealed that chestnut fruit has rich nutritive value: in addition to a high content of resistant starches, chestnuts provide a substantial source of vitamins, protein, minerals and essential fatty acids as well as polyphenols with antioxidant activity [3]. Furthermore, Chestnut fruit has medicinal value due to its many biological activities, including reduction of abdominal fat and serum cholesterol levels, inhibition of diseases caused by *Escherichia coli* (*E*. *coli*) and Streptomyces scabies, induction apoptosis of cervical cancer cells, and enhancement of gastrointestinal health. Thus, consumption of chestnuts is popular and recommended for human nutrition and health [4].

It should be noted that, unlike other nuts, which can be safely stored at room temperature, chestnuts maintain their health, turgor, and optimum commercial quality for only a relatively short time. This can be attributed to a number of factors, including the fact that chestnuts possess a porous epicarp with a high level of metabolic activity and a lack of lignification, as well as a relatively high moisture content, which ranges from 40% to 60% water, along with starch, which can all contribute to an increased risk of insect and mould infestation during storage [5,6,7]. The most common approach for extending chestnut shelf-life is cold storage, which inhibits physiological and metabolic changes and reduces chestnut decay loss and sprouting [8]. In order to maintain the optimal freshness for the high-value market, it is essential to store chestnuts at cold temperatures between 0 and 4 °C immediately following the harvest [9].

During the process of cold storage, plants develop the capacity to withstand freezing temperatures in response to low non-freezing temperatures, a phenomenon commonly referred to as cold acclimation [10,11]. In this process, plants accumulate compatible solutes, mainly carbohydrates such as raffinose, maltose, sucrose and fructose, to adjust osmotic potential in response to environmental stresses [12,13]. For example, the soluble sugar content was markedly elevated in cold-acclimated kale than in non-acclimated kale [14]. Additionally, tubers of potato (*Solanum tuberosum* L.) exhibited accelerated catabolism of starch into reducing sugars, including glucose and fructose, during low-temperature storage, a phenomenon known as cold-induced sweetening (CIS). It is unfortunate that the reducing sugars promote the non-enzymatic Maillard reaction, which results in an unsatisfactory dark colour and a bitter-tasting product. Furthermore, this process leads to the formation of acrylamide, a known carcinogen [15]. Regarding chestnuts, it is possible that cold storage can enhance the sweetness and flavour of the fruit when cooked or fried, compared to fruit that has not been stored. However, the mechanism behind this improvement in taste is still unclear, and the optimal duration for cold storage remains unknown.

Chestnuts are classified as recalcitrant seeds, characterised by a tendency to remain dormant in unfavourable winter conditions. This enables the seeds to survive until the following spring when they can germinate [16,17,18]. For seed dormancy release, one of the most commonly applied strategies is cold stratification, which is, in fact, a long-term cold storage process [19,20]. Various seeds need differential periods of cold stratification for physiological dormancy release. The seeds of *Euphorbia maculata* only need cold stratification (4 °C) for 15 days, but ginseng seeds need cold (2 °C) stratification for 3 months, while stratification (4 °C) for 120 days are suitable for complete dormancy release of *Glehnia littoralis* seeds [21,22]. A number of studies have demonstrated that, in addition to phytohormones, differentially expressed genes are also involved in the release of seed dormancy. Furthermore, these genes are found to be enriched in starch and sucrose, as well as in the plant energy metabolism pathway, and are associated with related physiological alterations [21,23,24]. Thus, there might be a logical connection between CIS and the dormancy release of chestnuts.

The aim of the present study was to ascertain the metabolic alterations in starch and soluble sugars and related hydrolytic enzyme activities, including amylase, sucrose synthase (SUS), sucrose phosphate synthase (SPS) and invertase in chestnuts stored for different periods at 4 °C as well as the dormancy release of chestnut seeds, and RNA-sequencing was employed for the purpose of identifying differentially expressed genes (DEGs) during the course of cold storage. By means of components assay, physiological and transcriptome analysis, we revealed the underlying mechanism for the taste improvement and the optimal time for consumption of chestnuts following cold storage and the correlation with dormancy release of chestnut seeds.

## 2. Materials and Methods

### 2.1. Materials

In this study, the Chinese chestnuts (Yanshan Zaofeng) utilised were collected in September 2021, at a mature developmental stage, within Qianxi County, Tangshan City, Hebei Province. The harvested fruit was collected and stored at 4 °C with 86–88% relative humidity for storage [25]. After storing for 1, 2 and 4 months, samples were randomly selected, the chestnuts were peeled, and kernels were ground in liquid nitrogen and frozen at −80 °C for further analysis. Three stages were clarified as follows: sweetening start stage (October, cold stored 1 month), sweetening completion stage (November, cold stored 2 months), and sweetening maintenance stage (January, cold stored 4 months) [26]. The amylopectin (99%) was procured from the Shanghai Macklin Biochemical Technology Co., Ltd. (Shanghai, China). The 3,5-dinitrosalicylic acid (DNS), sucrose and sodium hydroxide were procured from Sinopharm Chemical Reagent Co., Ltd. (Shanghai, China). The amylose, fructose and potassium phosphate were provided by Shanghai Yuanye Bio-Technology Co., Ltd. (Shanghai, China). Uridine diphosphate glucose (UDP-G) was purchased from Shanghai Aladdin Reagent Co., Ltd. (Shanghai, China). The chestnut cotyledons were used for transcriptome analysis; three parallel groups were set up for each group of experiments. Sequencing was completed using the Huada gene sequencing platform. All the other chemicals except amylopectin used were of analytical grade.

### 2.2. Determination of Sugar Contents by High-Performance Liquid Chromatography (HPLC)

In accordance with the methodology established by Zhao et al. [27], the sugar contents were determined with the following modifications. Ten millilitres of ultra-pure water was added to the peeled chestnut sample (10.0 g) and subsequently ground in a mortar. The mixture was transferred to a conical flask. An additional 10 mL of ultra-pure water was introduced to the mortar for rinsing purposes and was subsequently combined with the previous solution. Subsequently, the mixture was subjected to an ultrasonic bath for a period of 20 min, after which the volume was adjusted to 30 mL. Thereafter, the mixture was subjected to centrifugation at 8000× *g* for a period of 15 min, following which the supernatant was collected and filtered through a 0.45 µm filter.

Contents of sugars were detected by HPLC (Waters 1525, Shanghai, China) equipped with the carbohydrate chromatography columns (250 × 4.6 mm, 5 µm, ZORBAX Carbohydrate, USA) and refractive index detector. The mobile phase comprised 75% acetonitrile at a flow rate of 1.0 mL·min^−1^ with an injection volume of 10 μL.

### 2.3. Starch Contents Determination

Starch contents were determined using the dual-wavelength spectrophotometry method [28]. Amylose (25 mg·L^−1^) and amylopectin standard liquids (100 mg·L^−1^) were prepared to perform scanning from 400–800 nm in order to determine the detection wavelength and reference wavelength of each content. Chestnuts were peeled and mashed with a mortar and pestle. The oven-dried samples were weighed and sieved, 0.1 g of chestnut was mixed with 1 mL of ethanol, and then 9 mL of potassium hydroxide solution (1.0 mol·L^−1^) was added. After sealing the beaker with parafilm, the solution was immersed in a bath of boiling water for ten minutes. Once the solution had cooled to room temperature, the volume was added to the original scale with distilled water and fully dispersed. The aim of this step is to fully disperse the starch. 20 mL of the dispersion was degreased thrice with petroleum for 10 min. 2 mL of extract was transferred into a 100 mL beaker, followed by adding 6 mL of KOH (0.09 mol·L^−1^), 2 mL of acetic acid (1 mol·L^−1^) and 1 mL of iodine reagent. The total volume was adjusted to 50 mL with distilled water before standing at room temperature for 25 min. Absorbance was measured at 595 nm, 470 nm, 532 nm and 705 nm for each sample, with measurements taken in triplicate. A blank was prepared without adding a test solution.

### 2.4. Amylase Activity Determination

The peeled chestnuts (5.0 g) were pulverised using liquid nitrogen, mixed with citrate phosphate buffer (pH 5.6), and vortexed every 10 min at room temperature for a total of three times. Following this, the mixture was centrifuged at 5000× *g* for 10 min, and the resulting supernatant was retained. The residue was extracted again with the above buffer two times. All of the supernatants were transferred to a 100 mL volumetric flask, fixed with buffer and shaken well to obtain the amylase stock solution for activity determination of total amylase activity and alpha-amylase activity, as described by Guo et al. [29]. The β-amylase activity was determined by subtracting the α-amylase activity from the total amylase activity.

### 2.5. Sucrose Synthase and Acid Invertase Assays

The determination of sucrose synthase and acid invertase followed the method described by Wang et al. [30], with minor modifications made. Samples were grounded in liquid nitrogen, then mixed with potassium phosphate buffer (100 mM, pH 7.5, containing 5 mM MgCl_2_, 2.5 mM dl-dithiothreitol (DTT), 0.1% (*v*/*v*) TritonX-100 and 2% polyvinylpolypyrrolidone cross-linked (PPVP) at the ratio of 1:2 (*w*/*v*)). The homogenates were subjected to centrifugation at 10,000× *g* for 30 min at 4 °C, and then the supernatant fraction was added with ammonium sulfate to 80% saturation. Following a 6 h period of incubation at 4 °C, the mixture was subjected to centrifugation at 10,000× *g* for 20 min at 4 °C. The resulting pellets were collected and resolved in 20 mM potassium phosphate buffer (pH 7.5, containing 25 mM MgCl_2_) and dialysed overnight with changing buffer 3 times, and then the solution was kept at 4 °C for enzyme activity determination.

For the sucrose synthase assay, the reaction system contained HEPES-MP NaOH buffer (50 mM, pH 7.5), 15 mM MgCl_2_, 60 mM fructose, 25 mM UDP-glucose, and crude enzyme extract. The reaction mixture was incubated at 37 °C for 30 min, after which time it was terminated by adding 30% (*w*/*v*) 0.1 mL KOH and boiling for 5 min. The sucrose content was determined by employing the Anthrone-sulfuric acid technique.

The reaction system of acid invertase comprised 100 mM sucrose, sodium acetate-acetic acid (pH 5.0, 100 mM) and crude enzyme extract. After incubation at 37 °C for 1 h, the reaction was terminated by the transfer of the mixture to boiling water for a period of 5 min. The quantity of released glucose was determined by means of the 3,5-dinitrosalicylic acid method.

### 2.6. Transcriptome Sequencing

The reference genome (Genebank: GCA_000763605.2) information of chestnut in this study was obtained from NCBI (https://www.ncbi.nlm.nih.gov/ (accesses on 13 Jaunary 2022)) [31]. The sample was prepared in triplicate. The extraction of RNA was conducted using a plant total RNA extraction kit (Aidlab Biotechnologies Co., Ltd., Beijing, China) in accordance with the manufacturer’s instructions (with three biological replicates). The Illumina HiSeq 2000 sequencing platform was employed for the sequencing of total RNA, which was conducted by the BGI Company (Shenzhen, China, http://en.genomics.cn/ (accesses on 7 May 2022)). cDNA was synthesised with a HiScript ^®^II Q RT Super Mix for qPCR (+gDNA wiper) reverse transcription kit (Vazyme Biotechnology Co., Ltd., Nanjing, China), according to the manufacturer’s instructions. NanoDrop (Thermo Scientific, Waltham, MA, USA) was used to evaluate RNA purity and quantification [32]. The experimental procedure was conducted in triplicate. A differential expression analysis using DESeq2 was employed to identify genes that were differentially expressed [33]. Subsequently, a gene ontology (GO) enrichment analysis and a Kyoto Encyclopedia of Genes and Genomes (KEGG) enrichment analysis were conducted to ascertain which pathways were significantly enriched with respect to the differentially expressed genes, as compared to all annotated genes. The Benjamini and Hochberg approach was employed to adjust the resulting *p*-values in order to control the false discovery rate (FDR) [34]. A threshold of an adjusted *p*-value of ≤0.05 and a log fold change (FC) of ≥1.5 (|log_2_FC| ≥ 0.5849) was established to identify statistically significant differences in gene expression. The raw reads were submitted to the NCBI Sequence Read Archive (SRA) database and are accessible via the following accession number: PRJNA1081182.

### 2.7. Quantitative Reverse Transcription PCR (qRT-PCR) Validation

The relative expression levels of eight randomly selected genes and the key gene that exhibited differential expression in the chestnut cold-induced sweetening process were analysed by qRT-PCR. Primers for nine selected genes were designed using the Primer Premier 5.0 software (http://www.premierbiosoft.com (accessed on 24 June 2022)), and the primer information is presented in Table S1. The cDNA was synthesised using the HiScript^®^ II Q RT SuperMix for qPCR (+gDNA wiper) kit from Novozymes (Tianjin China). In accordance with the methodology proposed by Livak et al. (2001) [35], the internal control gene, β-actin, was employed as the reference gene. Polymerase chain reaction (PCR) amplification involved a pre-degeneration step at 95 °C for 30 s, denaturation at 95 °C for 5 s, and extension at 60 °C for 30 s, repeated for 40 cycles. The relative gene expression was calculated using the comparative Ct (2^−ΔΔCt^) method, with three replicates set for each gene [35].

### 2.8. Seed Dormancy Release Assay

Three replicates of 15 nuts of fruits cold stored for 1, 2 and 4 months were placed in a plastic basket; the baskets were then placed in a big plastic vent and air-way humidified by a humidifier to make suitable humidity for seeds to germinate in a room with air-conditioner to keep the temperature at 22 °C. Every 2 days the nuts were checked with seed radicle protrusion over 2 mm as the criterion of germination. The germination rate was calculated as the total number of germinated fruits divided by the total number of seeds in each treatment. The data were analysed using a ratio of the number of germinated seeds to the total number of seeds in each treatment, with a 10-day incubation period.

### 2.9. Statistical Analysis

The data were averaged from the three parallel experiments and are expressed as the mean ± standard deviation. The mean and variance were calculated using Microsoft Excel 2019, and significance analysis and Spearman correlation coefficient were calculated using SPSS 25.0. The significance level of difference was *p*-value < 0.05, and Origin 2 was used for graphing.

## 3. Results

### 3.1. Changes in Sugar Contents of Chestnut during Cold Storage

**Figure 1:**
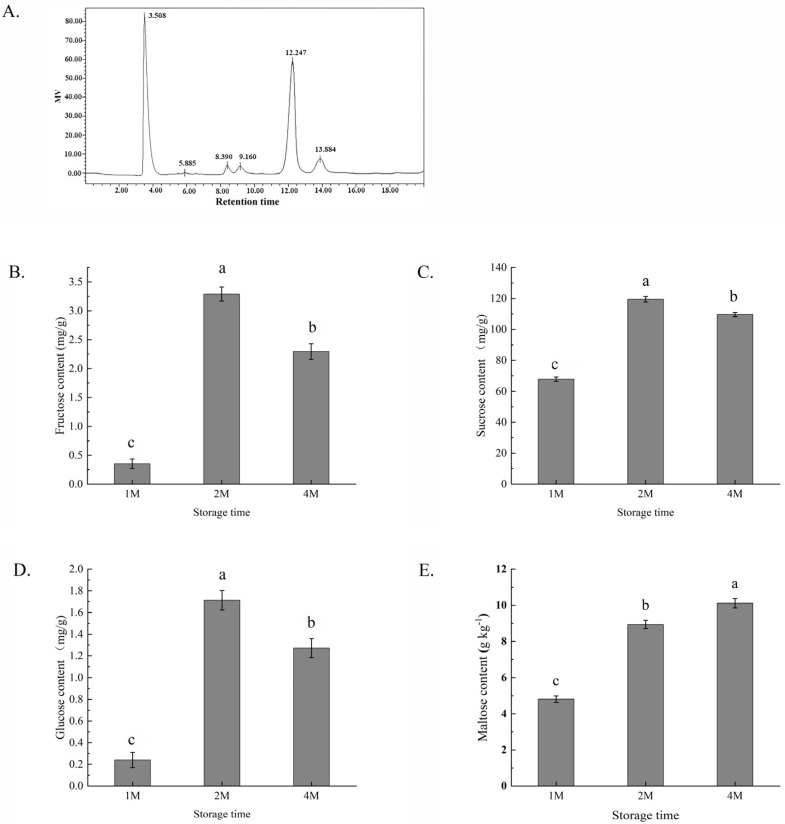
Profiles of several sugar contents in chestnuts measured by high-performance liquid chromatography (**A**) and changes in the contents of fructose (**B**), sucrose (**C**), glucose (**D**) and maltose (**E**) of chestnuts during cold storage for 1, 2 and 4 months. Fructose peaked at 8.390 min, glucose at 9.160 min, sucrose at 12.247 min and maltose at 13.884 min. Values in a column marked with a, b, c indicate significant differences between 1M, 2M and 4M samples (*p* < 0.05). In consideration of the magnitude of the group means, the most elevated value is designated as “a”, followed by “b”, with the least significant represented by “c”.

The composition of the sugars was determined by HPLC with a refractive index detector, employing a comparison with the corresponding component standards. The content of rhamnose was low during the whole storage period and sometimes below the detection threshold, so it was not analysed in this study. The peaks at 8.3, 9.1, 12.2 and 13.8 min were identified as fructose, glucose, sucrose and maltose, respectively (Figure 1A). All of the four sugar contents of chestnuts were low after one month of cold storage (in October), but a significant increase in the levels of all four kinds of sugars after 2 months of storage (in November). In 1M, the contents of glucose, fructose and sucrose demonstrated a slight decrease but still remained at high levels, and maltose levels were elevated. In all of the samples, sucrose was the main component.

### 3.2. Starch Contents Alternation in Chestnut Kenel during Cold Storage

The alterations in starch content observed in chestnuts during the three stages of development are illustrated in Table S2, and it can be observed that the starch content varied significantly and ranged from 44.43 to 31.59 % during cold storage. The major component of chestnut starch is amylopectin, and less with amylose. It is obvious that amylopectin content decreased while the amylose content was firstly decreased and then slightly increased with the increase of cold preservation time.

### 3.3. Changes in Enzyme Activities Related to Cold-Induced Sweetening

**Figure 2:**
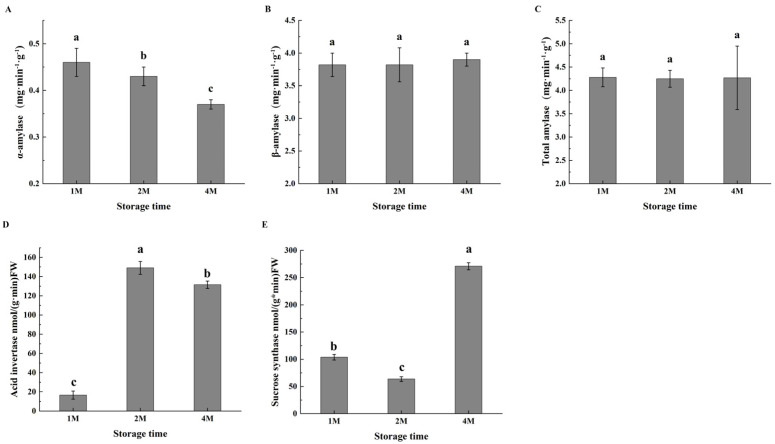
Changes in the activities of α-amylase (**A**), β-amylase (**B**), total amylase (**C**), acid invertase (**D**) and sucrose synthase (**E**) of chestnuts during cold storage. Values in a column marked with a, b, c indicate significant differences between 1M, 2M and 4M samples, which were stored for the same period of time (*p* < 0.05). In consideration of the magnitude of the group means, the most elevated value is designated as “a”, followed by “b”, with the least significant represented by “c”.

Changes in amylase activities associated with cold-induced sweetening were observed (Figure 2); while α-amylase activity decreased slightly during cold storage, β-amylase activity increased with the time of storage, with the level of about five times of α-amylase. The total amylase activity was the highest in 1M, then decreased slightly but still remained at a high level; the bioactivities of both amylases resulted in significant degradation of total starch in chestnuts (Table S2).

Given the accumulation of sucrose during the cold storage period, the activities of enzymes involved in sucrose metabolism were subsequently determined. During sucrose metabolism, acid invertase (AI) degrades sucrose to glucose and fructose. The AI activity was much low in October, then markedly increased in November, and maintained at a high level in January. As shown in Figure 2, sucrose synthase exhibited high activity in 1M, then decreased to some extent in 2M, but restored activity in 4M (Figure 2E).

### 3.4. Transcriptome Analyses in Chestnut during Cold Storage

#### 3.4.1. Differential Gene Expression Analysis

According to the taste performances of frying dried chestnuts and the sugar accumulation of chestnuts in the cold-induced sweetening process, three stages were clarified as follows: sweetening start stage (October, cold stored 1 month), sweetening completion stage (November, cold stored 2 months), and sweetening maintenance stage (January, cold stored 4 months). Chestnut cotyledons from three distinct stages of low-temperature storage were selected for RNA extraction and transcriptome sequencing, with three replicates of each group. Overall, a total of 4,101,300,000 raw reads were obtained from the nine samples, and 3,883,600,000 clean reads were retained following the removal of low-quality reads. The Q20% and Q30% of each sample were higher than 98% and 94%. A total of 58.25 Gb of clean reads was acquired, with an average of 6.47 Gb of clean reads per sample. Finally, a total of 29,895 genes were detected as expressed by transcriptome sequencing, of which 26,821 were known and 3074 were novel. A total of 90% or more of the clean reads could be successfully mapped to the reference genome, and the unique match rate was higher than 60%. These outcomes attest to the high quality of the sequencing data, which is suitable for subsequent analysis (Table S3).

Differentially expressed genes were screened based on *q* ≤ 0.05 and |log_2_ FC| ≥ 1 (Figure S1). In the three cold storage stages of chestnut, there were a total of 8363 DEGs between 2M and 1M, with 3682 upregulated and 4681 downregulated genes; 11417 DEGs between 4M and 2M with 6695 up-regulated and 4722 down-regulated, and 10210 DEGs between 4M and 1M with 5926 up-regulated and 4284 down-regulated (Figure S1A). A Venn diagram clearly showed the expression of differential genes between samples (Figure S1B).

#### 3.4.2. GO and KEGG Annotation and Enrichment Analysis of DEGs

The biological functions of the DEGs were subjected to further analysis using the GO and KEGG databases. The GO-annotated differentially expressed genes were primarily classified into three major categories: molecular function, cellular component, and biological process. We selected the top 20 GO terms that were most significantly (*q* ≤ 0.05) enriched in DEGs at the three time points of low-temperature storage. Results showed that the DEGs detected at the 1M, 2M and 4M time points were mainly enriched in intracellular (GO:0005622), cytoplasm (GO:0005737), organelle (GO:0043226), intracellular organelle (GO:0043229), nitrogen compound metabolic process (GO:0006807), membrane-bounded organelle (GO:0043227) and the intracellular membrane-bounded organelle (GO:0043231) projects, with intracellular enriched to the highest number of DEGs (Figure S2A). We also selected the top 20 KEGG pathways with significant DEG enrichment at the three time points of low-temperature storage, and the results showed that the most significant (q ≤ 0.05) DEGs were mainly concentrated in the ribosome (ko03010), RNA transport (ko03013), carbon metabolism (ko01200), protein processing in the endoplasmic reticulum (ko04141), spliceosome (ko03040), and biosynthesis of amino acids (ko01230). Two of the pathways, spliceosome and RNA transport showed the most genes to be enriched (Figure S2B).

#### 3.4.3. Sucrose Starch Metabolism Pathway-Related DEGs

**Figure 3:**
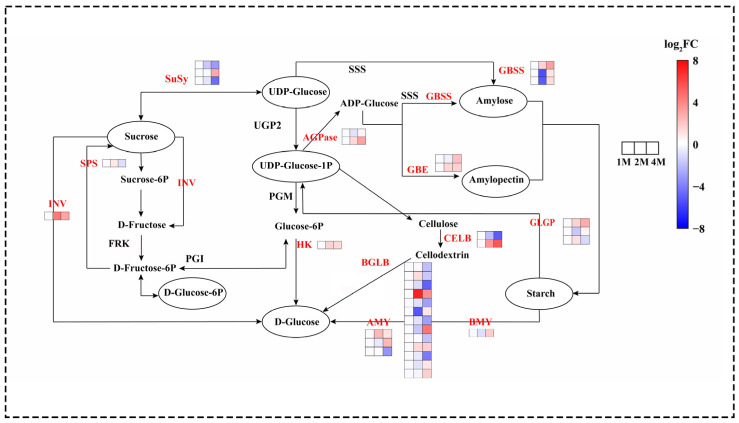
Heatmap of expression levels of DEGs of chestnut sucrose starch metabolism at different times during cold storage, generated based on log_2_ (fold change), where red represents a high level of expression and blue a low level. All three groups above were compared with 1M. The gene for INV is beta-fructofuranosidase. The gene for HK is hexokinase. The two genes for ADPase are glucose-1-phosphate adenylyltransferase. The three genes responsible for AMY are alpha-amylase. The gene for beta-amylase is BMY. The two genes for SBE are 1,4-alpha-glucan branching enzymes. The three genes for GBSS are granule-bound starch synthase. The three genes responsible for SuSy are sucrose synthase. The three genes responsible for GLGP are glycogen phosphorylase. The gene name of SPS is sucrose phosphate synthase. The two gene names of CELB are endoglucanase.

The differential expression of genes involved in sucrose starch metabolism was observed during distinct cold storage periods. The principal genes identified were those encoding the key enzymes involved in starch biosynthesis and degradation, sucrose metabolism and maltose conversion (Figure 3).

A total of 85 genes were identified as exhibiting significant differential expression in the sucrose starch synthesis pathway (Figure 2). The analysis revealed that genes associated with starch synthesis, including soluble starch synthase (SSS), 1,4-α-glucan branching enzyme (GBE), granule-bound starch synthase (GBSS) and ADP-glucose pyrophosphorylase (AGPase) exhibited increased expression during the cold storage phase. α-amylase (AMY), β-amylase (BMY), and glycogen phosphorylase (GLGP) are enzymes involved in the degradation of starch. During cold storage, the expression of 2 AMY genes, 1 BMY gene, and 6 GLGP genes increased. Sucrose synthase (SUS) and sucrose phosphate synthase (SPS) are key to the process of sucrose synthesis. The expression of the 1 SPS gene first increased at 2M, followed by a decrease at 4M, and the expression of the 2 SuSy genes was continuously decreased at the refrigerated stage. Invertase (INV), hexokinase (HK), and beta-D-glucosidase (BGLB) are enzymes involved in the hydrolysis and conversion of glucose and fructose. In this study, most of the genes encoding INV, HK and BGLB showed an up-regulation in expression during cold storage (Figure 2 and Table S3).

#### 3.4.4. KEEG Enrichment and DEGs Involved in Phytohormones Biosynthesis and Signal Transduction Pathway

In order to gain insight into the roles played by phytohormones in the process of CIS, an analysis of genes involved in phytohormone synthesis and signal transduction pathways was conducted using the KEGG database (Figure 3, Tables S5 and S7).

A variety of phytohormones comprise a complex network that regulates dormancy. Genes involved in the synthesis, metabolism, and signalling of hormones, including abscisic acid (ABA), gibberellins (GAs), indole acetic acid (IAA), cytokinin (CTK), ethylene (ETH), brassinosteroid (BR), and jasmonic acid (JA), exhibited significant expression during the progression of seed dormancy release (Table S6).

We screened for significantly differentially expressed genes in phytohormone synthesis and signal transduction based on *p* ≤ 0.05 as a criterion. The majority of genes involved in jasmonic acid (JA) biosynthesis exhibited increased expression levels throughout the storage period, including two lipoxygenase (LOX) genes, one 12-oxo-phytodienoic acid reductase (OPR) gene and one allene oxide cyclase (AOC) gene. The expression level of the gene encoding ent-kaurene oxidase (KO), which is involved in GA biosynthesis, was down-regulated and then up-regulated, and most of the gibberellin 2-oxidase (GA2ox) genes were down-regulated. In the ETH biosynthesis pathway, three S-adenosylmethionine synthetase (SAMS) genes were down-regulated from the 1M stage to the 4M stage. The three genes of 1-aminocyclopropane-1-carboxylic acid oxidase (ACO) are consistently up-regulated. The genes involved in ABA biosynthesis mostly presented a down regulatory tendency, including one gene coding for 9-cis-epoxycarotenoid dioxygenase (NCED) and six genes coding for ABA2 during the entire storage period.

**Figure 4:**
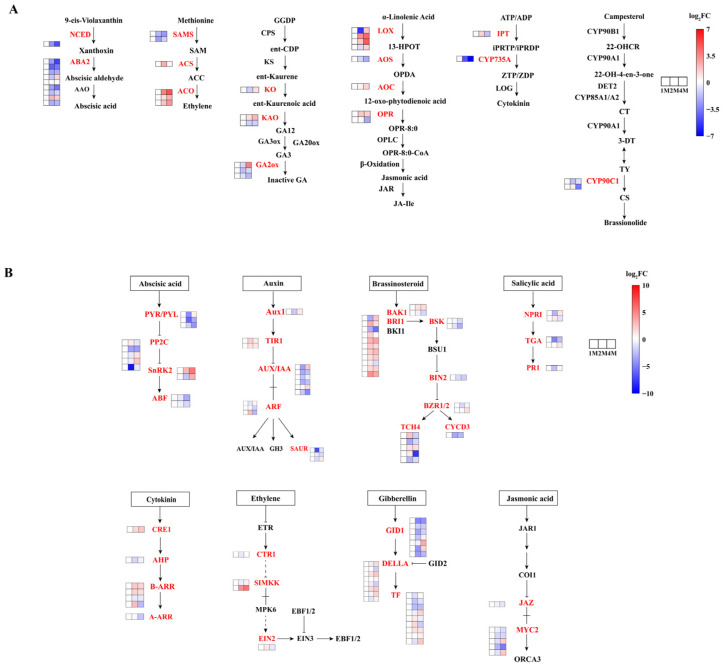
Heatmap of expression levels of DEGs of chestnut phytohormone biosynthesis (**A**) and chestnut phytohormone signal transduction (**B**) at different times during cold storage, generated based on log_2_ (fold change), where red represents a high level of expression and blue a low level. All three groups above were compared with 1M. The gene for NCED is 9-cis-epoxycarotenoid dioxygenase. The nine genes responsible for ABA2 are xanthoxin dehydrogenase. The gene for KO is ent-kaurene oxidase. The two genes for KAO are ent-kaurenoic acid monooxygenase. The three genes for GA2ox are gibberellin 2beta-dioxygenase. The two genes responsible for SAMS are the S-adenosylmethionine synthetase. The gene responsible for ACS is the 1-aminocyclopropane-1-carboxylate synthase. The three gene names of ACO are aminocyclopropanecarboxylate oxidase. The fourth gene name of LOX is lipoxygenase. The gene name of AOS is hydroperoxide dehydratase. The three gene names of OPR are 12-oxophytodienoic acid reductase. The gene for AUX1 is auxin influx carrier (AUX1 LAX family). The two genes for TIR1 are transport inhibitor response 1. The six genes for AUXIAA are auxin-responsive protein IAA. The three genes for ARF are auxin response factors. The three genes for SAUR are the SAUR family protein. The gene for CRE1 is arabidopsis histidine kinase 2/3/4 (cytokinin receptor). The gene for AHP is histidine-containing phosphotransfer protein. The four genes for B-ARR are two-component response regulator ARR-B family. The gene for A-ARR is a two-component response regulator ARR-A family. The seven genes for GID1 are auxin gibberellin receptor GID1. The eight genes for DELLA are DELLA proteins. The three genes for PYRPYL are the abscisic acid receptor PYR/PYL family. The five genes for PP2C are protein phosphatase 2C. The two genes for SnRK2 are threonine–protein kinase SRK2. The two genes for ABF are ABA-responsive element binding factors. The gene for EIN2 is ethylene-insensitive protein 2. The two genes for BAK1 are brassinosteroid insensitive 1-associated receptor kinase 1. The eleven genes for BRI1 are protein brassinosteroid insensitive 1. The two genes for BSK are BR-signaling kinase. The gene for BIN2 is protein brassinosteroid insensitive 2. The two genes for BZR12 are brassinosteroid resistant 1/2. The five genes for TCH4 are xyloglucan:xyloglucosyl transferase TCH5. The gene for CYCD3 is cyclin D3. The gene for JAZ is a Jasmonate ZIM domain-containing protein. The five genes for MYC2 are transcription factor MYC2. The two genes for NPR1 are regulatory protein NPR1. The two genes for TGA are transcription factor TGA. The gene for PR-1 is pathogenesis-related protein 1. The arrows in the figure refer to activation and the dotted lines refer to a change of state.

The DEGs involved in phytohormone signalling are presented in Figure 4B and Table S7. In the ABA signal transduction pathway, two abscisic acid receptors (PYR/PYL) genes, three protein phosphatase 2C (PP2C) genes and two ABA-responsive element binding factor (ABF) genes were down-regulated in three stages, and two sucrose non-fermenting-1-related protein kinase 2 (SnRK2) genes, one PYR/PYL gene and three PP2C genes were first down-regulated then up-regulated. In the SA signalling pathway, one transcription factor TGA (TGA) gene was down-regulated, and one gene was up-regulated. Among the genes related to ETH signal transduction pathways, one copper transport protein (CTR1) gene and one mitogen-activated protein kinase kinase 4/6 (SIMKK) gene were down-regulated and then up-regulated. As for GA signal transduction, two DELLA proteins (DELLAs) genes and four gibberellin-insensitive dwarf1 (GID1) genes were down-regulated, and two DELLA genes (CMV_006654 and CMV_016382) were up-regulated. Two auxin receptor transporter inhibitor response protein 1 (TIR1) genes in IAA signal transduction were up-regulated, and four AUX/IAA genes, two small auxin-up RNAs (SAUR) genes and one ARF gene were down-regulated. The expression of three myelocytomatosis factors (MYC2) genes and one Jasmonate ZIM-domain protein (JAZ) gene responsible for JA signal transduction were down-regulated. For the BR signalling pathway, one BRI1 Associated Receptor Kinase 1 (BAK1) gene, seven brassinosteroid insensitive 1 (BRI1) genes, one xyloglucan:xyloglucosyl transferase TCH5 (TCH4) gene and one brassinazole-resistant factor (BZR1/2) gene were up-regulated, and one BAK1, two BR-signaling kinase (BSK) genes, one brassinosteroid insensitive 2 (BIN2) gene and one cyclin D3 (CYCD3) gene were down-regulated.

#### 3.4.5. Differentially Expressed Transcription Factors in Chestnuts during Cold Storage

**Figure 5:**
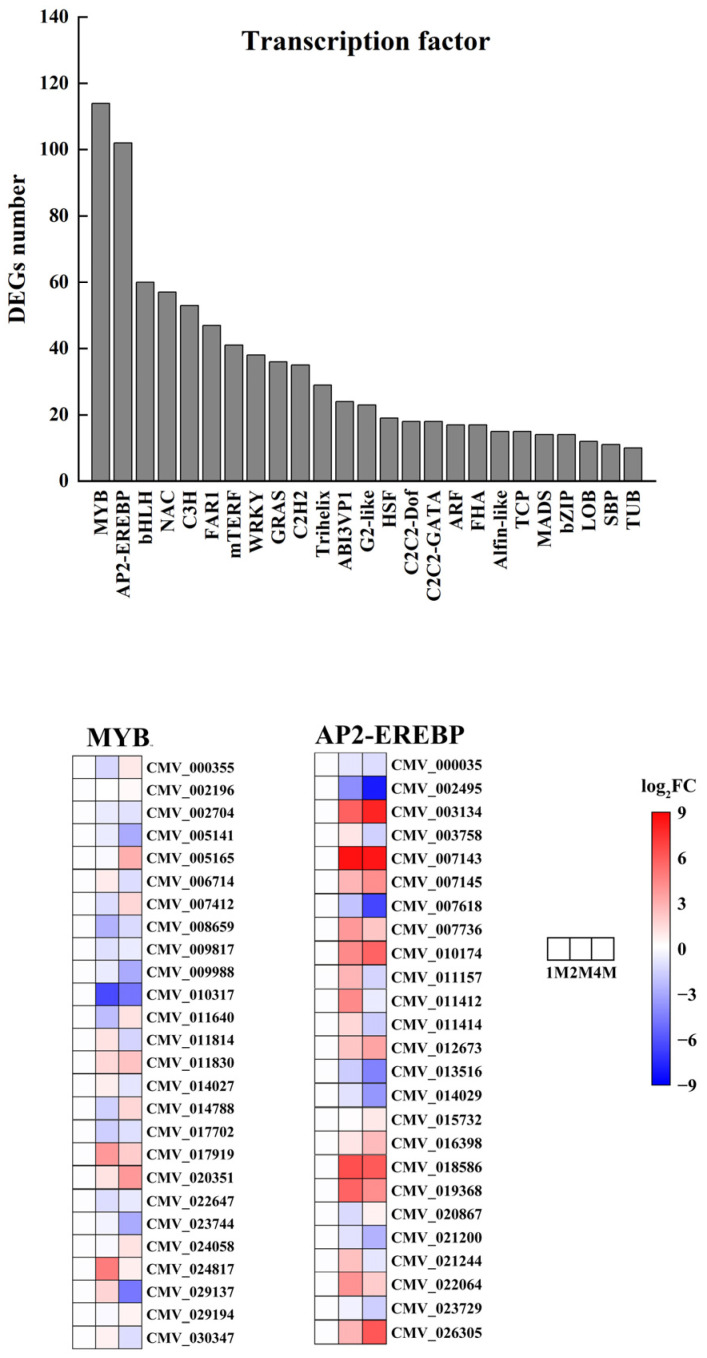
Number of transcription factor differential expressed genes in chestnut under cold storage and Heatmap of relative expression levels of typical MYB and AP2-EREBP transcription factors.

To explore the impact of storing chestnuts at low temperatures on transcription factors, we conducted an analysis of transcription factors for the differentially expressed genes in 1M, 2M and 4M. The results demonstrated that a total of 984 transcription factors exhibiting differential expression were annotated to the three refrigeration stages from 57 distinct transcription factor families. The transcription factor families in question are primarily comprised of MYB, AP2-EREBP, bHLH, NAC, C3H, FAR1, mTERF, WRKY, GRAS, C2H2, Trihelix, ABI3VP1, G2-like, HSF, C2C2-Dof, C2C2-GATA, ARF, FHA, Alfin- like, TCP, MADS, bZIP, LOB, SBP, and TUB. The first two families had the highest number of differential genes, with the MYB family containing 114 members and the AP2-EREBP family containing 102 members (Figure 5 and Table S8).

We analysed the expression levels of all MYB, AP2/ERF-ERF family genes in the 1M vs. 2M, 2M vs. 4M and 1M vs. 4M groups. The MYB family contained 2, 49 and 42 up-regulated genes and 85, 47 and 45 down-regulated genes in the three groups, and the AP2/ERF-ERF family contained 1, 48 and 44 up-regulated genes and 72, 37, 38 and 45 down-regulated genes in the three groups (Table S4).

### 3.5. Quantitative RT-PCR (qRT-PCR) Analysis

To validate some of the differentially expressed genes (DEGs), quantitative real-time polymerase chain reaction (q-PCR) was conducted. A total of nine genes were randomly selected for q-PCR analysis. As illustrated in Figure S3, the outcomes were largely concordant with the RNA-seq data, thereby substantiating the reliability of the RNA-seq data.

### 3.6. Effects of Cold Storage Time on Chestnut Germination

**Figure 6:**
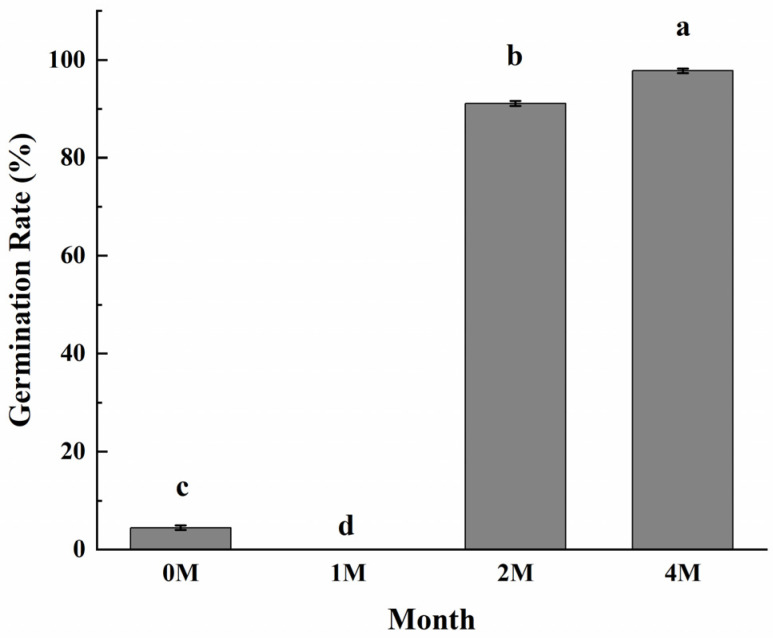
Effects of different storage periods on seed sprouting rates of chestnuts stored at 0 °C. Values in a column marked with a, b, c indicate significant differences between 1M, 2M and 4M samples, which were stored for the same period of time (*p* < 0.05). In consideration of the magnitude of the group means, the most elevated value is designated as “a”, followed by “b”, then “c”, with the least significant represented by “d”.

Since chestnuts have dormancy, and cold storage and high humidity are the means for dormancy release, we wonder if the sweetening process induced by cold storage is related to the dormancy breakage of fruits. The germination results (Figure 6) clearly showed that chestnuts cold stored for 2 months sprouted vigorously at a rate that reached 91.1%, and the germination rate of the fruits stored for 4 months reached nearly 97.8%, while the fruits did not sprout with only 1 month of cold storage. The results demonstrated that cold storage for 2 months is enough for chestnut to release dormancy. 

## 4. Discussion

In China, stir-frying chestnuts is one of the popular consumption types. The typical taste quality characteristics of fried nuts are soft, glutinous, sweet and rich in flavours [36]. However, newly harvested chestnuts are meally and lack sweetness; the fruits should be stored at low temperatures for one or two months to obtain the best taste of fried chestnuts. This process, traditionally, is called enhancing sweetness. Previous studies have demonstrated that the application of low storage temperatures facilitates the acceleration of starch-sugar conversions and elevates the sugar content observed in sweet potatoes, potatoes and lily bulbs post-cold storage [37]. To avoid possible freezing injury on chestnuts, in this study, cold storage at 0 °C was applied for investigation of cold-induced sweetening effects.

Since chestnut fruits are seeds for completing generation, a substantial quantity of starch is stored in the parenchyma cells of the cotyledon. These cells provide the nutrients and energy for seed dormancy maintenance and release for germination under low-temperature conditions [38]. On the other hand, low temperature also functioned as a stress signal, eliciting responses against the chilling injury [39]. The metabolism of carbohydrates is highly active in low-temperature environments, leading to alterations in nutrient content and, consequently, an impact on food quality [40]. The most significant constituent of chestnuts is starch, which plays a pivotal role in influencing the texture characteristics of fried chestnuts. The mealiness of potato varieties is generally correlated with high starch contents, and the content of starch, as well as the ratio of amylose to amylopectin, contributes to the crisp texture of the lotus rhizome [41]. Furthermore, post-harvest starch degradation in response to cold stress altered the texture of buttercup squash and lily bulbs from farinose viscous to crisp and juicy [42,43]. In this study, the contents of starch decreased from 444.3 mg/g to 354.9 mg/g after one and two months of cold storage, with amylopectin accounting for most of the alternations (Table S2). The gene encoding GBSS (CMV_024168, CMV_027527) was found to be significantly up-regulated and then significantly down-regulated in all three phases, resulting in a decrease and then a slight increase in the content of amylose. The gene encoding SBE (CMV_001728) exhibited the highest expression in 1M when amylopectin content was elevated, while in 4M, the enzyme catalysed the conversion of amylose into amylopectin, preparing for the release of dormancy. Similar results were observed in lily bulbs [43]. This is partly due to degradation by α-amylase (AMY) and β-amylase (BAM). As we determined, the activities of both of the enzymes were high and stable in cold conditions for three periods, with BAM activities nearly five times that of AMY (Figure 2), which indicated that BAM contributed to cold-induced sweetening of chestnuts. Several reports revealed that specific transcription of genes encoding AMY isoforms was induced by low temperatures, including StAmy23 in potato tubers and Amy8 in apple fruits [44,45]. In our study, the RNA sequencing data demonstrated that the genes encoding AMY were moderately expressed in chestnut fruits after one and two months of cold storage, which was consistent with the activities of the enzyme. BAM catalyses the release of β-maltose and is believed to be the major pathway of starch degradation in Arabidopsis and other organisms [46,47]. Some isoforms of BAM are considered cold-responsive, such as BAM3 contributes to leaf starch degradation in mesophyll cells of Arabidopsis at night and under cold stress [46,47], a substantial increase in β-amylase expression and an abundant accumulation of reducing sugars in potato tubers cooled to 3–5 °C [48]. These results suggest that β-amylase may play a significant role in potato cold-induced sweetening. We also observed that gene encoding BAM (CMV_013993) was slightly down-regulated in October and November and up-regulated in January (Figure 3), which may be related to the fact that BAM has remained highly active and supported the idea of BAM as the causal agent for starch degradation in cold-induced sweetening of chestnuts.

It has been widely accepted that the soluble sugar composition and concentration (sucrose, maltose, glucose and fructose) are major contributors to fruit sweetness, and the changes in concentrations of soluble sugars altered the quality of fruit stored at low temperatures [49]. In this study, sucrose was the main soluble sugar, followed by fructose and glucose in chestnuts under cold storage; similar phenomena were also observed in kiwi berries and lily bulbs [50]. Growing evidence demonstrates that a higher level of sucrose is necessary to enhance fruit quality, delay fruit senescence, and prevent chilling injury [51,52]. Glucose and fructose have also been associated with cold resistance in fruits [53]. Sucrose synthesis and dissociation into glucose and fructose in various cellular compartments are regulated by sucrose synthase, sucrose phosphate synthase, acid invertase and neutral invertase [54]. The present study showed that the activities of sucrose synthase and acid invertase were maintained at a high level, along with a high concentration of sucrose and constant levels of fructose and glucose. RNA-seq results showed that the gene encoding invertase (CMV_019870) was up-regulated constantly, but the expression level was lower than those of the genes encoding sucrose synthase (CMV_005375, CMV_006399) and SPS (CMV_020186), which in turn enhanced accumulation of sucrose. Thus, our results would support the idea that the high energy level maintained fruit quality and improved fruit chilling tolerance through energy homeostasis, ROS scavenging and osmotic maintenance [55,56].

Starch degradation and sucrose accumulation under long-term cold storage are common phenomena in potato tubers [57], bulbs of Gladiolus [58], Liliaceae [59] and onions [60], which are organs for propagation with the character of chilling-induced dormancy breakage before sprouting. Several pieces of research revealed that the transformation of a substantial quantity of starch into sucrose not only furnishes the energy and carbon source necessary for the release of dormancy but also as an important signal [61]. As chestnuts are seeds and after 2 months of cold storage, sucrose increased and reached the peak and decreased slightly; then, the fruits were prone to sprouting, which indicates that the dormancy had been released at this time (Figure 1). Phytohormones regulate bud dormancy in plants [62]. The release of dormancy and promotion of bud burst are induced by gibberellins, cytokinins, and brassinosteroids [63,64]. Conversely, ethylene and ABA promote the maintenance of dormancy [65,66]. Transcriptome analysis in chestnuts demonstrated that enzymes and transcriptional factors involved in the biosynthesis of GA, BR and JA were highly expressed after two months of cold storage, while the genes involved in ABA and ethylene biosynthesis mostly presented a down-regulation tendency; the expression profiles of genes involving in phytohormone signal transduction also support the roles of phytohormones in dormancy release (Figure 4). Furthermore, the alternation of starch and sucrose metabolism under cold conditions also influences dormancy release. The potato Stvinv knock-outlines, which encodes vacular acid invertase, showed prolonged dormancy in association with the lower sugar units accumulation, and 35S:: stvinv tubers (overexpression line) showed shorter dormancy duration, associated with increased sugar units [57] while silencing of potato alpha-amylase 23 (StAmy23) also inhibited dormancy release in cv. Solara [67]. BAM plays a dominant role in starch degradation. Inhibition of BAM by carvone treatment results in a dormant state in sweet potato [68]. The gene expression analysis indicated that the gene encoding BMA exhibited a slight down-regulation at 2M, with BMA activity remaining almost unchanged. At this time point, the maltose content demonstrated a slight increase. At 4M, the breakage of the dormant state led to a significant up-regulation of BMA expression and an increase in maltose content. These results indicated that the cold induced sweetening and phytohormone alternations during cold storage are interconnected during dormancy release, as various amounts of transcriptional factors were differentially expressed during cold storage. Our results on the chestnut sprouting assay that cold storage for 2 months was enough for the dormancy release of chestnuts (Figure 6) further supported the idea that cold-induced sweetening is correlated with the dormancy release of chestnuts.

Combined with the physiological observation and transcriptome data, we verified that the taste improvement of chestnuts after cold storage was due to the partial conversion of starch to sucrose accumulation, which is an extremely complex process, with enzymes and genes involved in regulation far more than these presented here. The detailed regulation of sweetening in chestnut fruits, including cold signal perception and transduction, the interactions between phytohormones and starch-sucrose conventions, as well as their role in dormancy release and against chilling, are needed for further investigation in detail. 

## 5. Conclusions

Cold-induced sweetening of chestnuts stored at low temperatures was due to the accumulation of sucrose, which was accompanied by starch reduction and activation of starch and sucrose metabolism-related enzymes. Transcriptome data further showed that differentially expressed genes were enriched in starch and sugar metabolism pathways, phytohormone biosynthesis and signal transduction, as well as transcriptional factors, which were interrelated with cold stress tolerance and seed dormancy release of chestnuts.

## Data Availability

The original contributions presented in the study are included in the article/Supplementary Material, further inquiries can be directed to the corresponding author.

## Supplementary Materials

The following supporting information can be downloaded at https://www.mdpi.com/article/10.3390/foods13172822/s1.
