# Supplementary material for: Transcriptome Analysis Reveals the Mechanism of Cold-Induced Sweetening in Chestnut during Cold Storage
Source: Foods. 2024 Sep 5;13(17):2822. doi: 10.3390/foods13172822 (PMC11394792; doi:10.3390/foods13172822)
Supplement: Supplementary file 1 [file foods-13-02822-s001.zip › foods-3141784 - supplementary.pdf]

Transcriptome analysis reveals the mechanism of cold-induce sweetening in chestnut during cold storage

Chun Zhan †, Ruqi Jia †, Shuzhen Yang, Meihong Zhang and Litao Peng *

Supplementary material

Table S1. Primers of target gene for qRCR

Gene ID	Forward Primer (5'-3')	Reverse Primer (5'-3')
CMV_010876	TGCTGGAACTCGCTTGTTTC	CGAGCAAGGTGACGATTGAG
CMV_003994	AAGTTGGGTTGCCTGTTGAC	TAAATTTTGCCACACCCCGG
CMV_001728	TCCAACTTAAGGTGGTGGCT	CCATTGCAAGGCGGTATTCA
CMV_004905	GCTTCTGATGCCGGTAAACA	TGAAGGAAAACGTGGCATCG
CMV_001080	TCGGTGCCTTCCAGTGTTAT	CATCTGCGAGTACCAGCCT
CMV_019180	ACTGCTGAACTTCTCTGGCT	CAATCCCAAGGTTCCGCAAT
CMV_005529	CACCAAAGCATATGAGGCCC	TCTGGGATGCAGAGAGTTGG
CMV_005375	TGCCCCTGAAACCCCTTATT	AGAAATGTCTCGAGGGTGCA
CMV_015650	CCTTCGTGGCATCCTCTACT	TACACCAGCCCGAAAGTCAT
CMV_011669	ACTGCAGGCTGTGACATACT	ACCCTGTACCTTCACCATGG
Actin	ATTCACGAGACCACCTACA	TGCCACAACCTTAATCTTCAT

Table S2. Changes in starch content of chestnuts during cold storage at 4°C

Storage time	Amylose (%)	Amylopectin (%)	Total starch (%)
1M	5.17±0.30 ^{a}	39.26±0.58 ^{a}	44.43±0.23^{a}
2M	4.68±0.33 ^{c}	30.82±0.24 ^{b}	35.49±0.61^{b}
4M	4.92±0.41 ^{b}	26.67±0.69 ^{c}	31.59±0.47^{c}

Values are presented as the mean ± SD. Values in a column marked with a, b, c, indicate significant differences between 1M, 2M and 4M samples at the same storage time ($p < 0.05$).

Table S3. Summary of transcriptome sequencing results.

Sample	Total Raw Reads (M)	Total Clean Reads (M)	Total Clean Bases (Gb)	Q20 (%)	Q30 (%)	Clean Reads Ratio (%)	Total Mapping (%)	Uniquely Mapping (%)
BL10X1	45.57	43.51	6.53	98.19	94.8	95.47	90.89	63.5
BL10X2	45.57	43.37	6.5	98.28	94.92	95.16	91.11	65.6
BL10X3	45.57	43.48	6.52	98.3	94.94	95.41	91.55	66.12
BL11X1	45.57	43.3	6.5	98.27	94.86	95.01	91.42	67.17
BL11X2	45.57	43.2	6.48	98.26	94.85	94.78	91.37	67.43
BL11X3	45.57	43.08	6.46	98.27	94.86	94.52	91.37	67.22
BL1X1	45.57	42.94	6.44	98.27	94.9	94.22	91.22	66.98
BL1X2	45.57	42.54	6.38	98.31	94.98	93.34	90.83	67.1
BL1X3	45.57	42.94	6.44	98.37	95.16	94.23	90.43	66.73

Table S4. MYB and AP2-EREBP family differential genes in three three cold storage stages.

Group	1M vs 2M		2M vs 4M		1M vs 4M	
	Down-regulated	Up-regulated	Down-regulated	Up-regulated	Down-regulated	Up-regulated
MYB	85	2	47	49	45	42
AP2-EREBP	72	1	37	48	38	44

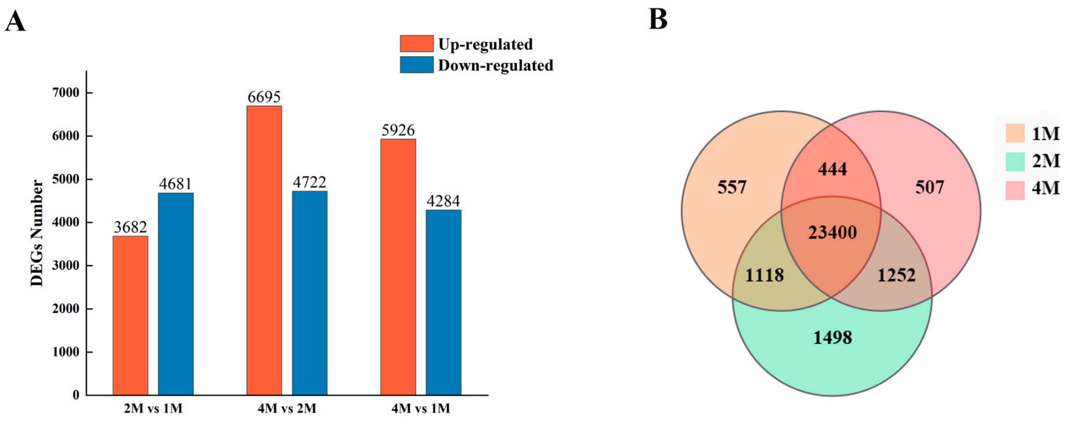

Fig. S1 Differential gene expression during the cold storage stage of chestnut. (A) Number of differential genes in chestnut between three cold storage time groups. (B) Venn diagram showing the different expressed unigene between treatments with different cold storage times. 1M, 2M and 4M denote the 1st, 2nd and 4th months of chestnut refrigeration, respectively.

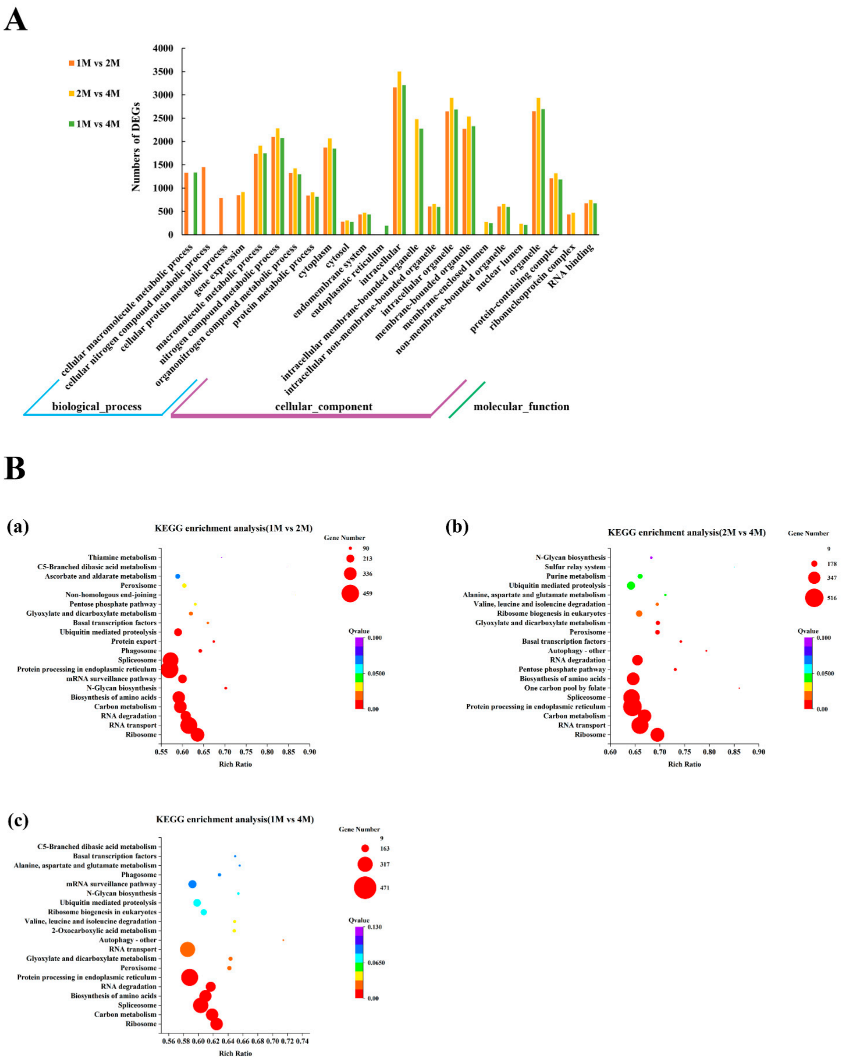

Fig. S2 GO annotation and KEGG enrichment analysis. (A) The top 20 GO terms for the differentially expressed between 1M vs 2M, 2M vs 4M and 1M vs 4M in chestnut under refrigeration. (B) The top 20 KEGG pathways for the differentially expressed between 1M vs 2M, 2M vs 4M and 1M vs 4M in chestnut under refrigeration:（a）KEGG pathway enrichment analysis of DEGs between 1M and 2M;（b）KEGG pathway enrichment analysis of DEGs between 2M and 4M;（c）KEGG pathway enrichment analysis of DEGs between 1M and 4M.

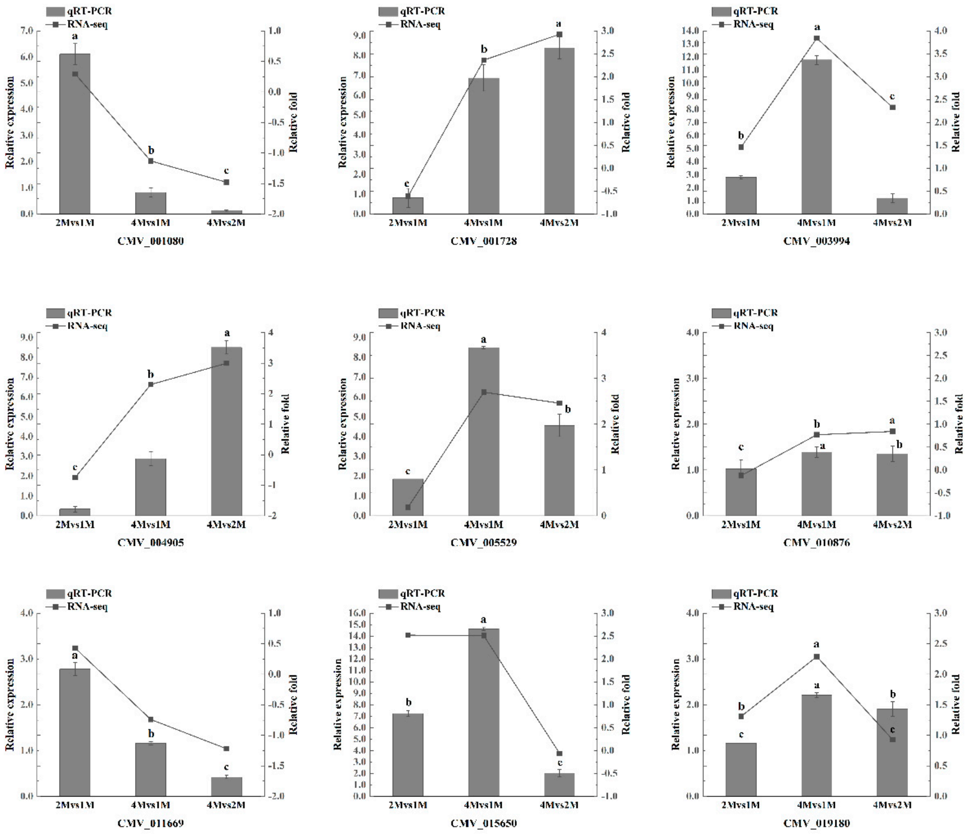

Fig. S3 Validation of the expression patterns of differentially expressed genes (DEGs) selected from the RNA-seq analysis by qRT-PCR. Values in a column marked with a, b, c, indicate significant differences between 1M, 2M and 4M groups at the same storage time ($p < 0.05$). 1M, 2M and 4M denote the 1st, 2nd and 4th months of chestnut refrigeration, respectively.
